# Identified risk factors for co-infection in hospitalised children infected with adenovirus in Hangzhou

**DOI:** 10.1017/S0950268822000565

**Published:** 2022-03-30

**Authors:** Qun Lao, Ning Han, Haipeng Pan, Ming Zhan, Yidong Wu, Shiyong Zhao, Yuzhu Jia

**Affiliations:** 1 Department of Radiology, Hangzhou Children's Hospital, Hangzhou 310014, PR China; 2 Department of Radiology, Zhejiang Xiaoshan Hospital, Hangzhou 310014, PR China; 3 Clinical Laboratory, Hangzhou Children's Hospital, Hangzhou 310014, PR China; 4 Department of Infection, Hangzhou Children's Hospital, Hangzhou 310014, PR China; 5 Department of Radiology, Tongde Hospital of Zhejiang Province, Hangzhou 310014, PR China

**Keywords:** Adenovirus infections, China (D002681), confounding factors, epidemiologic (D015986), human (D000258)

## Abstract

This study aimed to describe the clinical manifestations of adenovirus infections and identify potential risk factors for co-infection with chlamydia, viruses and bacteria in hospitalised children from Hangzhou, China. From January to December 2019, the characteristics of hospitalised children infected with adenovirus at Hangzhou Children's Hospital and Zhejiang Xiaoshan Hospital were collected. The clinical factors related to co-infection with chlamydia, viruses and bacteria were assessed using multivariate logistic regression analyses. A total of 5989 children were infected with adenovirus, of which 573 were hospitalised for adenovirus infection. The severity of adenovirus respiratory infection was categorised as follows: mild (bronchiolitis, 73.6%), moderate (bronchopneumonia, 17.6%) or severe (pneumonia, 8.8%). Of the 573 children who were hospitalised, 280 presented with co-infection of chlamydia, viruses or bacteria, while the remaining 293 had only adenovirus infection. Multivariate stepwise logistic regression analyses indicated that elevated ferritin was associated with an increased risk of chlamydia co-infection (odds ratio (OR) 6.50; 95% confidence interval (CI) 1.56–27.11; *P* = 0.010). However, increased white blood cell (WBC) count was associated with a reduced risk of viral co-infection (OR 0.84; 95% CI 0.75–0.95; *P* = 0.006). The study indicated that co-infection with chlamydia could be affected by elevated ferritin levels. WBC levels could affect viral co-infection in hospitalised children infected with adenovirus.

## Introduction

Human adenoviruses (HAdVs) are double-stranded non-enveloped DNA viruses belonging to the genus *Mastadenovirus*, family Adenoviridae. These viruses cause various clinical manifestations, including acute respiratory infections, gastroenteritis, conjunctivitis, cystitis and meningoencephalitis [1]. HAdV infections account for 5–7% of respiratory illnesses in paediatric patients and 1–7% of those in adults [2, 3]. Populations susceptible to HAdV infection include those aged <5 years, those who live in densely populated areas and immunocompromised populations [4]. HAdVs are classified into seven species (A–G) and include more than 60 serotypes [5]. The clinical manifestations and severity of the disease are significantly correlated with the serotype [6].

Outbreaks of respiratory tract HAdV have been recently reported in Jiangsu and Taiwan provinces of China, Korea, Singapore and Malaysia [7–9]. Although most cases presented with mild to moderate disease, life-threatening diseases were also detected. This was especially the case in immunocompromised populations [10]. Children who have co-infection with other pathogens are especially vulnerable to developing severe disease [11]. A previous study attempted to identify the risk factors for disease severity and found that the duration of hospitalisation, lymphocyte count and lactate dehydrogenase (LDH) levels could all affect disease severity. Independent risk factors for co-infection were not identified [12], the present study aimed to explore the clinical manifestations of HAdV infection in children and the potential risk factors for co-infection with chlamydia, viruses and bacteria in hospitalised children infected with HAdV.

## Methods

### Patient inclusion and exclusion criteria

Data for 5989 children with HAdV infections who presented at Hangzhou Children's Hospital and Zhejiang Xiaoshan Hospital from January to December 2019 were collected. Among these, 573 children who were hospitalised for HAdV infection were recruited to explore the risk factors for co-infection with chlamydia, viruses and bacteria. All recruited patients were <14.0 years. These patients displayed positive results in HAdV tests with a reverse transcription polymerase chain reaction assay. The exclusion criteria were as follows: (1) congenital heart disease, (2) congenital pulmonary disease, (3) malignancy, (4) severe organ dysfunction, (5) infection in other organs and (6) use of corticosteroids within 1 week. The Institutional Review Board of Hangzhou Children's Hospital and Zhejiang Xiaoshan Hospital approved this study (No. 201803). All enrolled patients’ parents or guardians provided written informed consent for research purposes.

### Variable measurements

The participants’ data were collected from electronic medical records. Clinical information, laboratory results and radiological findings of 573 patients were extracted. These data included the season of onset, fever duration, hospital stay, serum amyloid A levels, procalcitonin levels, C-reactive protein (CRP) levels, white blood cell (WBC) count, neutrophil count, lymphocyte count and the severity of HAdV respiratory infection. Levels of alanine aminotransferase (ALT), aspartate aminotransferase (AST), creatine kinase (CK), creatine kinase isoenzyme (CK-MB), LDH and ferritin were also extracted. Disease severity was based on the American Thoracic Society's guidelines for the management of community-acquired pneumonia, which categorises respiratory disease as mild (bronchiolitis), moderate (bronchopneumonia) or severe (pneumonia) [13]. A Digital Diagnostic TH system with a Philips DR machine was used to obtain the chest radiographs. The automatic exposure control was adopted to set mAs and Kv values according to the patients’ age as follows: 58–65 Kv/1–2 mAs for patients aged 1–6 years or 65–80 Kv/2–3 mAs for those aged over 6 years. The GE OPTIMA 540 system was used to obtain CT images with the following parameters: 16 rows; tube voltage, 80–100 kVp for children aged 1–5 years and 120 kVp for children aged >5.0 years; tube current, 10–300 mA; pitch, 1.375:1; collimation, 40 mm; layer thickness and spacing, 5 mm.

### Definition of co-infection

The co-infections were grouped into those caused by chlamydia, viruses or bacteria. No co-infection was considered as a control. Co-infection caused by chlamydia was determined by enzyme-conjugate immunoassay, while virus co-infection was tested using swabbed with a nasopharyngeal sample collection kit. Moreover, fungal and bacterial infections were assessed through blood cultures before using antibiotics. Children who showed positive results for HAdV and co-infection of chlamydia, a virus or a bacterium were regarded as displaying co-infection and were divided into chlamydia, viral or bacterial infected groups. The mixed co-infection was defined as infected two types of chlamydia, viruses or bacteria.

### Statistical analysis

The collected characteristics of children were presented as median (quartile) and number (percentage) for continuous and categorical variables, respectively. The differences in characteristics among the chlamydia, viral or bacterial co-infection and control groups were assessed by Wilcoxon and *χ*^2^ tests. Multivariate logistic regression analysis was performed. These were conducted to estimate multivariate adjusted odds ratios (ORs) and 95% confidence intervals (CIs) for the risk factors related to subsequent co-infection with chlamydia, viruses or bacteria. Multivariate analyses were performed using both the full model and stepwise regression approach. All reported *P* values were two-sided, and *P* values <0.05 were considered statistically significant. Statistical analysis was performed using SPSS version 22 software (SPSS, Chicago, Illinois, USA).

## Results

### Epidemiologic characteristics of infected children

**Fig. 1.**
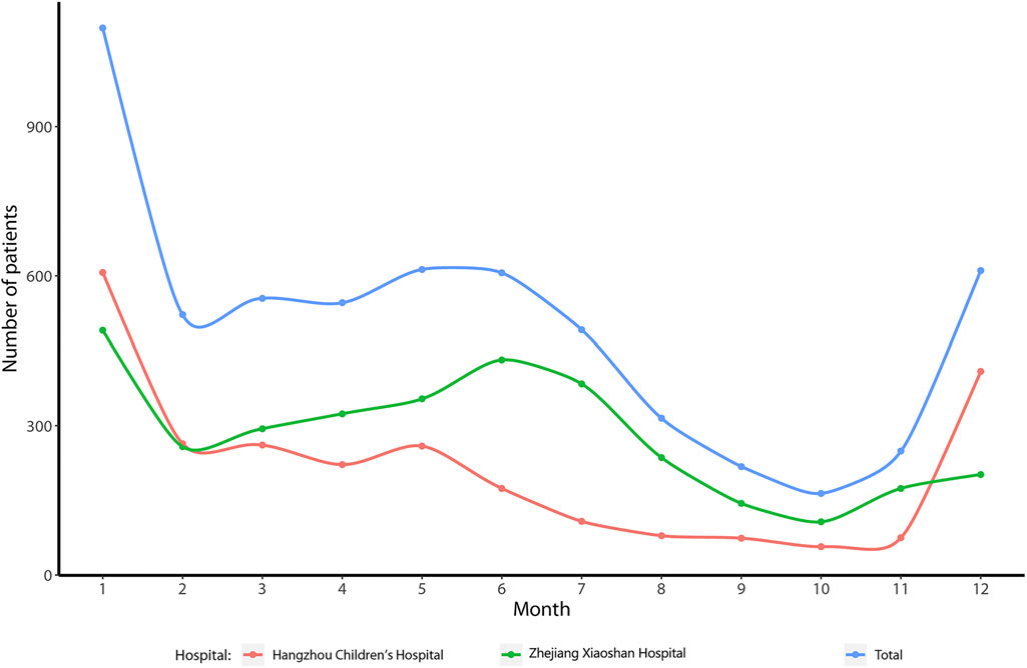
The epidemic curve for children with HAdV infections.

The prevalence of HAdV infection according to months of disease onset is shown in Figure 1. The peak months of disease onset were December, January, May and June. A dip in infections was observed in August, September, October and November. Of 5989 children with HAdV infection, the mean age was 4.17 years. Most patients were aged 6.0 months or older. A total of 1746 children were aged 6.0 months to 2.0 years, 1930 were aged 2.0–4.0 years and 2278 were aged 4.0 years or older. Only 35 patients were aged 6.0 months or younger.

### Characteristics of infected children and disease severity

A total of 573 children were hospitalised for HAdV respiratory infection, of which 73.6% showed bronchiolitis, 17.6% displayed bronchopneumonia and the remaining 8.8% exhibited severe pneumonia. Of these patients, 280 showed co-infection, while the remaining 293 were infected only with HAdV. One child presented with brachial plexus nerve injury. There were significant differences among the groups in terms of hospital stay (*P* = 0.002), WBC count (*P* < 0.001), neutrophil count (*P* = 0.002) and ferritin levels (*P* = 0.023). However, there were no significant differences in the month of onset (*P* = 0.250), fever duration (*P* = 0.174), serum amyloid A level (*P* = 0.954), procalcitonin level (*P* = 0.569), CRP level (*P* = 0.516), lymphocyte count (*P* = 0.487), ALT (*P* = 0.431), AST (*P* = 0.675), CK (*P* = 0.932), CK-MB (*P* = 0.660) and LDH (*P* = 0.846) (Table 1).

**Table 1.** Baseline characteristics of recruited children infected with adenovirus

		Co-infection				
Variable	Overall (*n* = 573)	Control (*n* = 293)	Chlamydia (*n* = 161)	Virus (*n* = 41)	Bacterial (*n* = 78)	*P* value
Onset of months	5.00 (2.00, 8.00)	5.00 (3.00, 8.00)	5.00 (1.00, 8.00)	6.00 (3.00, 8.00)	5.00 (1.00, 7.00)	0.250
Onset of months (category)						0.435
January	105 (18.32)	31 (10.58)	46 (28.57)	2 (4.88)	26 (33.33)	
February	51 (8.90)	39 (13.31)	5 (3.11)	6 (14.63)	1 (1.28)	
March	41 (7.16)	30 (10.24)	5 (3.11)	5 (12.20)	1 (1.28)	
April	33 (5.76)	16 (5.46)	9 (5.59)	1 (2.44)	7 (8.97)	
May	66 (11.52)	34 (11.60)	18 (11.18)	6 (14.63)	8 (10.26)	
June	64 (11.17)	33 (11.26)	16 (9.94)	6 (14.63)	9 (11.54)	
July	60 (10.47)	32 (10.92)	18 (11.18)	3 (7.32)	7 (8.97)	
August	31 (5.41)	16 (5.46)	10 (6.21)	3 (7.32)	2 (2.56)	
September	23 (4.01)	12 (4.10)	3 (1.86)	1 (2.44)	7 (8.97)	
October	23 (4.01)	12 (4.10)	9 (5.59)	0 (0.00)	2 (2.56)	
November	26 (4.54)	13 (4.44)	8 (4.97)	2 (4.88)	3 (3.85)	
December	50 (8.73)	25 (8.53)	14 (8.70)	6 (14.63)	5 (6.41)	
Fever duration (days)	4.00 (3.00, 7.00)	4.00 (2.00, 6.00)	5.00 (3.00, 7.00)	4.00 (3.00, 6.00)	5.00 (3.00, 7.00)	0.174
Hospital stay (days)	8.00 (6.00, 13.00)	7.00 (5.00, 11.00)	9.00 (6.00, 15.00)	7.00 (5.00, 13.00)	8.50 (6.00, 14.00)	0.002
Serum amyloid A	134.40 (97.50, 152.90)	135.10 (95.30, 153.30)	129.90 (103.80, 151.50)	137.40 (81.55, 154.8)	137.95 (98.00, 152.7)	0.954
Procalcitonin	0.61 (0.17, 3.78)	0.64 (0.17, 3.51)	0.35 (0.15, 3.83)	0.65 (0.17, 4.74)	1.21 (0.17, 3.66)	0.569
C-reactive protein	18.29 (7.37, 33.40)	19.15 (7.84, 35.05)	14.06 (6.57, 30.83)	22.00 (6.48, 36.18)	19.95 (7.97, 33.14)	0.516
White blood cell	11.23 (9.18, 13.40)	11.42 (9.35, 13.46)	10.68 (8.95, 13.21)	9.51 (8.22, 10.97)	12.05 (10.34, 14.26)	<0.001
Neutrophil count	6.15 (4.40, 7.73)	6.31 (4.72, 7.82)	5.93 (4.24, 7.78)	4.89 (3.34, 5.92)	6.92 (4.56, 8.33)	0.002
Lymphocyte count	3.10 (2.14, 4.20)	3.13 (2.16, 4.38)	3.02 (1.98, 4.06)	3.13 (2.44, 4.08)	3.46 (2.33, 4.20)	0.487
ALT	30.00 (15.00, 49.00)	29.00 (14.00, 47.00)	33.00 (17.00, 49.00)	22.00 (12.00, 48.00)	36.00 (16.50, 49.50)	0.431
AST	30.00 (16.00, 42.00)	31.00 (18.00, 42.00)	27.00 (15.00, 41.00)	30.00 (12.00, 45.00)	31.00 (16.00, 40.50)	0.675
CK	99.00 (64.00, 148.00)	98.00 (64.00, 151.50)	104.00 (67.00, 145.00)	95.00 (67.00, 144.00)	91.00 (50.50, 148.00)	0.932
CK-MB	21.00 (16.00, 35.00)	21.00 (15.50, 34.50)	21.00 (17.00, 31.00)	22.00 (17.00, 35.00)	23.00 (16.50, 40.00)	0.660
Lactate dehydrogenase	342.00 (276.00, 440.00)	341.50 (271.00, 441.50)	345.50 (284.50, 438.50)	338.00 (275.00, 421.00)	334.50 (269.00, 415.00)	0.846
Ferritin > 1500	11 (1.94)	3 (1.04)	8 (5.00)	0 (0.00)	0 (0.00)	0.023

### Risk factors for co-infection with chlamydia

The results of multivariate logistic regression based on the full model revealed that the onset months of February (OR 0.12; *P* < 0.001), March (OR 0.11; *P* < 0.001), June (OR 0.32; *P* = 0.010), July (OR 0.41; *P* = 0.043) and December (OR 0.37; *P* = 0.028) were associated with a reduced risk of chlamydia co-infection as compared with disease onset in January. Multivariate stepwise logistic regression revealed that February (OR 0.10; *P* < 0.001), March (OR 0.10; *P* < 0.001), May (OR 0.35; *P* = 0.011), June (OR 0.28; *P* = 0.003), July (OR 0.35; *P* = 0.012), September (OR 0.22; *P* = 0.031) and December (OR 0.35; *P* = 0.017) were associated with a reduced risk of chlamydia co-infection as compared with disease onset in January. However, both full model (OR 6.50; *P* = 0.010) and stepwise (OR 6.48; *P* = 0.013) logistic regression found a ferritin level >1500 was associated with an increased risk of chlamydia co-infection (Table 2).

**Table 2.** Multivariable adjusted analyses for the risk of co-infection of chlamydia, virus and bacterial

Variable	Full model		Stepwise regression^a^	
	OR (95% CI)	*P* value	OR (95% CI)	*P* value
*Co-infection (chlamydia vs control)*				
Onset of months (January)	Ref	–	Ref	–
February	0.12 (0.04–0.36)	<0.001	0.10 (0.04–0.30)	<0.001
March	0.11 (0.03–0.39)	<0.001	0.10 (0.03–0.34)	<0.001
April	0.54 (0.19–1.56)	0.254	0.42 (0.15–1.12)	0.083
May	0.48 (0.20–1.15)	0.100	0.35 (0.16–0.79)	0.011
June	0.32 (0.13–0.76)	0.010	0.28 (0.12–0.65)	0.003
July	0.41 (0.18–0.97)	0.043	0.35 (0.15–0.79)	0.012
August	0.60 (0.22–1.67)	0.330	0.51 (0.19–1.36)	0.180
September	0.25 (0.06–1.04)	0.057	0.22 (0.05–0.87)	0.031
October	0.71 (0.23–2.21)	0.553	0.63 (0.21–1.88)	0.407
November	0.42 (0.14–1.31)	0.136	0.35 (0.12–1.03)	0.056
December	0.37 (0.15–0.90)	0.028	0.35 (0.14–0.82)	0.017
Fever duration (days)	0.98 (0.91–1.06)	0.607		
Hospital stay (days)	1.03 (0.98–1.08)	0.194		
Serum amyloid A	1.00 (1.00–1.00)	0.689		
Procalcitonin	0.98 (0.92–1.05)	0.559		
C-reactive protein	0.99 (0.98–1.01)	0.403		
White blood cell	1.05 (0.87–1.27)	0.579	0.95 (0.90–1.01)	0.115
Neutrophil count	0.89 (0.72–1.10)	0.291		
Lymphocyte count	0.85 (0.67–1.09)	0.213		
ALT	1.01 (0.99–1.02)	0.298		
AST	0.99 (0.98–1.00)	0.172		
CK	1.00 (1.00–1.00)	0.970		
CK-MB	1.00 (0.99–1.00)	0.326		
Lactate dehydrogenase	1.00 (1.00–1.00)	0.231		
Ferritin>1500	6.48 (1.49–28.29)	0.013	6.50 (1.56–27.11)	0.010
*Co-infection (virus vs control)*				
Onset of months (January)	Ref	–	Ref	–
February	1.60 (0.28–9.32)	0.600	1.78 (0.33–9.69)	0.507
March	1.31 (0.19–9.15)	0.787	1.59 (0.24–10.49)	0.632
April	0.51 (0.04–7.10)	0.616	0.62 (0.05–7.69)	0.708
May	1.10 (0.18–6.92)	0.917	1.43 (0.25–8.26)	0.690
June	1.85 (0.32–10.57)	0.488	1.73 (0.32–9.48)	0.528
July	0.94 (0.14–6.43)	0.947	0.97 (0.15–6.39)	0.979
August	2.23 (0.31–16.15)	0.427	2.16 (0.32–14.70)	0.431
September	0.93 (0.07–12.59)	0.959	1.00 (0.08–12.49)	0.997
October	0.00 (0.00–11 × 10^273^)	0.971	0.00 (0.00–17 × 10^293^)	0.974
November	1.21 (0.14–10.39)	0.863	1.53 (0.19–12.31)	0.690
December	2.46 (0.43–14.18)	0.315	2.23 (0.40–12.33)	0.357
Fever duration (days)	0.96 (0.85–1.09)	0.560		
Hospital stay (days)	1.00 (0.93–1.09)	0.941		
Serum amyloid A	1.00 (0.99–1.01)	0.585		
Procalcitonin	1.00 (0.96–1.04)	0.912		
C-reactive protein	1.00 (0.98–1.02)	0.875		
White blood cell	0.67 (0.44–1.01)	0.058	0.84 (0.75–0.95)	0.006
Neutrophil count	1.23 (0.78–1.93)	0.369		
Lymphocyte count	1.57 (0.95–2.60)	0.077		
ALT	1.00 (0.98–1.02)	0.762		
AST	0.99 (0.97–1.01)	0.275		
CK	1.00 (1.00–1.00)	0.901		
CK-MB	1.00 (0.98–1.01)	0.625		
Lactate dehydrogenase	1.00 (1.00–1.00)	0.833		
Ferritin > 1500	0.00 (0.00-I)	0.983	0.00 (0.00–>999)	0.983
*Co-infection (bacterial vs control)*				
Onset of months (January)	Ref	–	Ref	–
February	0.03 (0.00–0.23)	< 0.001	0.03 (0.00–0.25)	0.001
March	0.00 (0.00–26 × 10^168^)	0.946	0.00 (0.00–23 × 10^175^)	0.949
April	0.46 (0.12–1.70)	0.245	0.48 (0.16–1.44)	0.190
May	0.38 (0.13–1.14)	0.083	0.26 (0.10–0.71)	0.008
June	0.33 (0.12–0.92)	0.033	0.34 (0.13–0.89)	0.028
July	0.22 (0.07–0.70)	0.010	0.24 (0.08–0.70)	0.009
August	0.15 (0.03–0.78)	0.025	0.17 (0.03–0.84)	0.030
September	0.50 (0.13–1.83)	0.293	0.60 (0.17–2.03)	0.409
October	0.24 (0.04–1.28)	0.094	0.27 (0.05–1.38)	0.114
November	0.17 (0.03–0.89)	0.036	0.19 (0.04–0.93)	0.041
December	0.19 (0.06–0.65)	0.008	0.24 (0.08–0.75)	0.014
Fever duration (days)	1.00 (0.90–1.11)	0.947		
Hospital stay (days)	1.01 (0.95–1.08)	0.793		
Serum amyloid A	1.00 (0.99–1.00)	0.248		
Procalcitonin	0.99 (0.95–1.04)	0.751		
C-reactive protein	1.01 (0.99–1.02)	0.479		
White blood cell	1.34 (1.08–1.67)	0.009	1.01 (0.94–1.08)	0.858
Neutrophil count	0.71 (0.56–0.92)	0.008		
Lymphocyte count	0.79 (0.59–1.07)	0.124		
ALT	1.01 (0.99–1.02)	0.280		
AST	1.00 (0.98–1.02)	0.987		
CK	1.00 (1.00–1.00)	0.380		
CK-MB	1.00 (1.00–1.01)	0.492		
Lactate dehydrogenase	1.00 (1.00–1.00)	0.937		
Ferritin > 1500	0.00 (0.00–1.00)	0.978	0.00 (0.00–>999)	0.979

a Stepwise, *α*_inclusion_ = 0.05, *β*_exclusion_ = 0.10

### Risk factors for co-infection with viruses

The multivariate stepwise logistic regression found that elevated WBC levels were associated with a reduced risk of viral co-infection (OR 0.84; *P* = 0.006). No other significant indicators for co-infection with HAdV were observed (Table 2).

### Risk factors for co-infection with bacteria

The results of multivariate logistic regression based on the full model found that the onset months of February (OR 0.03; *P* < 0.001), June (OR 0.33; *P* = 0.033), July (OR 0.22; *P* = 0.010), August (OR 0.15; *P* = 0.025), November (OR 0.17; *P* = 0.036), December (OR 0.19; *P* = 0.009) and an elevated neutrophil count (OR 0.71; *P* = 0.008) were associated with a reduced risk of bacterial co-infection. Moreover, elevated WBC levels were associated with an increased risk of bacterial co-infection (OR 1.34; *P* = 0.009). The multivariate stepwise logistic regression suggested that the onset months of February (OR 0.03; *P* = 0.001), May (OR 0.26; *P* = 0.008), June (OR 0.34; *P* = 0.028), July (OR 0.24; *P* = 0.009), August (OR 0.17; *P* = 0.030), November (OR 0.19; *P* = 0.041) and December (OR 0.24; *P* = 0.014) were associated with a reduced risk of bacterial co-infection (Table 2).

## Discussion

The onset of HAdV in children remains controversial. The results of this study based on 5989 cases found that the peak periods of disease onset were in December, January, May and June. However, a dip in infections was observed in August, September, October and November. This result suggests that the onset of HAdV is significantly correlated with climatic factors, and the potential reason for this could be seasonal variability of the co-infecting pathogens. Moreover, the most frequent age of children infected with HAdV was 6.0 months or older. Furthermore, the risk of chlamydia co-infection could be affected by the month of onset and ferritin levels >1500. In addition, elevated WBC levels were significantly associated with a lower viral co-infection risk. Finally, the onset month, neutrophil count and WBC count could affect the risk of bacterial co-infection.

In this study, we noted that 73.6% of the children with respiratory disease presented with bronchiolitis. Their clinical manifestations were non-specific, including symptoms such as cough, runny nose and fever. Of the remaining patients, 17.6% presented with bronchopneumonia and 8.8% presented with severe pneumonia. The clinical manifestations of these cases included persistent high fever, dry cough, shortness of breath and hypoxemia. These symptoms were associated with a high incidence of mortality. These results are consistent with the findings of a previous study conducted in Korea [9]. Furthermore, the fever duration ranged from 0 to 13 days. The mean temperature fluctuated from 37.4 to 39.4 °C. This was not consistent with the study conducted by Xie *et al*. [14]. They noted that the temperature fluctuated from 40.0 to 40.9 °C in most children, the disease duration ranged from 3.0 to 14.0 days, and most cases entered the recovery period after 2.0 weeks.

Most cases presented with mild symptoms in the early stage and 73.6% of the children displayed bronchiolitis. The imaging characteristics in these cases included increased and thickened main veins in the lungs and a higher frequency of interstitial reticular shadows in the lower lobes of both lungs. Moreover, the characteristics of the moderate to severe cases included multiple clusters of consolidated shadows in both lungs, ‘centripetal’ lesions that were primarily found in the middle and inner lungs, enhanced fusion foci, additional large foci, added emphysema, extra lung texture, fewer round foci, less pulmonary bullae and few pleural effusions. These were associated with rapid progression, and multiple clusters of consolidated shadows in both lungs. Although the cases with moderate to severe symptoms presented with ‘centripetal’ lesions, they showed no obvious irregularity in the distribution and morphology of the bronchial tree. Finally, we did not observe positive cases of encephalitis. This indicated that HAdV infections did not involve the central nervous system (CNS). These characteristics could differentiate HAdV infections from A/H1n1 infections. This is because A/H1n1 tends to be distributed along the central bronchial tree with an intact bronchial tree shape. The H1N1 virus can easily invade the CNS, yielding positive images of the CNS. It may even cause necrotising encephalitis.

This study found that elevated ferritin levels were associated with an increased risk of chlamydia co-infection in hospitalised children infected with HAdV. The potential reason for this could be that ferritin not only represents iron reserves, but also serves as an inflammatory marker [15]. Moreover, we noted that elevated WBC levels were associated with a lower risk of viral co-infection and a higher risk of bacterial co-infection. The reason for this could be that WBC also serves as an inflammatory marker, which could affect bacterial co-infection. Thus, viral co-infection could not be reflected by WBC levels. Interestingly, we noted that an elevated neutrophil count was associated with a lower risk of bacterial co-infection. This was inconsistent with previous studies [16–18] and could be explained by the distribution of neutrophil counts in hospitalised children infected with HAdV. Therefore, elevated ferritin levels in hospitalised children infected with HAdV was considered as high-risk of chlamydia co-infection, while elevated WBC level was regarded as high-risk of bacterial co-infection.

Several limitations of this study should be acknowledged: (1) because this study was a hospital-based study and designed as retrospective, which can suffer from selection bias that is often associated with hospital-based retrospective studies; (2) the data available in the current study were based on electronic medical records, while the background therapies were not addressed; (3) stratified analyses based on the characteristics of patients were not conducted owing to the small number of patients in each group; and (4) the serotypes of the adenovirus, which could affect the clinical manifestations and severity of the disease, were not addressed in this study.

## Conclusions

This study describes the epidemiologic and clinical manifestations of HAdV infection in children. The risk of co-infection with chlamydia, viruses and bacteria could be affected by the month of onset, neutrophil count, WBC count and ferritin levels in hospitalised children infected with HAdV. Further prospective studies should be conducted to construct a predictive model for the prognosis of hospitalised children infected with HAdV.

## Data Availability

The datasets generated during and/or analysed during the current study are available from the corresponding author on reasonable request.
